# The Association between Tooth Loss and Insulin Resistance Mediated by Diet Quality and Systemic Immunoinflammatory Index

**DOI:** 10.3390/nu15235008

**Published:** 2023-12-04

**Authors:** Yaqi Hao, Shaoru Li, Shaojie Dong, Lin Niu

**Affiliations:** 1 Key Laboratory of Shaanxi Province for Craniofacial Precision Medicine Research, College of Stomatology, Xi’an Jiaotong University, Xi’an 710004, China; hyq3117328003@stu.xjtu.edu.cn (Y.H.); dongshaojie@xjtu.edu.cn (S.D.); 2 Clinical Research Center of Shaanxi Province for Dental and Maxillofacial Diseases, Xi’an 710004, China; 3 Experimental Teaching Center, School of Public Health, Xi’an Jiaotong University Health Science Center, Xi’an 710061, China; lishaoru@xjtu.edu.cn; 4 Department of Prosthodontics, College of Stomatology, Xi’an Jiaotong University, Xi’an 710004, China

**Keywords:** tooth loss, insulin resistance, HEI-2015 score, systemic immune-inflammation index

## Abstract

(1) Background: Both tooth loss and diabetes have high global prevalence, and both have a significant influence on patients’ general health and quality of life. Previous research has indicated a possible connection between tooth loss and diabetes, but it has been unclear whether tooth loss has an effect on the development of diabetes and how it affects it. We aim to investigate the relationship between insulin resistance (IR) and tooth loss and examine how the systemic immune-inflammation index (SII) level and diet quality mediate it. (2) Methods: The cross-sectional study data were obtained from the National Health and Nutrition Examination Survey (NHANES). After describing and comparing baseline data, we used regression models to evaluate the relationship between IR and tooth loss, diet quality and tooth loss and IR, SII and tooth loss and IR. Furthermore, we applied bootstrapping to test the mediation effect of diet quality and SII between tooth loss and IR. Diet quality is reflected by the HEI (Healthy Eating Index)-2015 score. (3) Results: The total number of subjects included was 8197, with 3861 individuals belonging to the IR group (HOMA-IR ≥ 2.5) and 4336 in the non-IR group (HOMA-IR < 2.5). In the model with all covariates adjusted, tooth loss in the fourth quartile was found to be positively correlated with an increase in HOMA-IR (OR = 1.301; 95% confidence interval (CI) = [1.102, 1.537]; *p* < 0.001) compared to the first quartile; tooth loss in the fourth quartile correlated with the HEI-2015 score compared to the first quantile (β = −0.121, 95% CI = [−4.839, −2.974], *p* < 0.001); and the highest number of tooth loss was found to have a significant effect on SII (β = 0.032; 95%CI = [1.777, 47.448]; *p* < 0.05). Compared to average diet quality, best diet quality acts as a safeguard against elevated HOMA-IR (OR = 0.776; 95% CI = [0.641, 0.939]; *p* < 0.01); inadequate diet quality is a risk factor (OR = 1.267; 95%CI = [1.138, 1.411]; *p* < 0.001) conversely. Meanwhile, it can be seen that compared with the first quantile of SII, the highest score is significantly correlated with the higher incidence of IR (OR = 1.363; 95%CI = [1.179, 1.575]; *p* < 0.001). Diet quality and SII played a partial mediating role in the relationship between HOMA-IR and tooth loss, and the mediating effect ratio for the total effect value was 4.731% and 4.576%, respectively. The mediating effect of SII and diet quality in the association of the relationship between HOMA-IR and tooth loss both was 0.003 (95%CI = [0.001, 0.004]). (4) Conclusions: Our study revealed the relationship between IR and tooth loss, and further explored the mediating role of SII and diet quality between the number of missing teeth and IR, emphasizing that improving diet quality and reducing SII can effectively prevent and treat IR and related diseases. It provides new theoretical support for the study of IR mechanisms and new ideas and approaches to deal with related diseases.

## 1. Introduction

Insulin resistance (IR) is the weakening of the biological effect of the body to insulin, including the decrease in sensitivity and responsiveness to insulin, thought to be the primary cause of type 2 diabetes mellitus (T2DM) [1]. In addition, IR is also linked to a variety of diseases, including non-alcoholic fatty liver, metabolic syndrome, atherosclerosis, cognitive impairment, endothelial dysfunction, and polycystic ovary syndrome [2]. Although the physiological mechanism of IR is not yet well established, understanding the factors that induce it is important for the prophylaxis and treatment of IR and related disorders.

According to the WHO Global Oral Health Status Report (2022), the global average incidence of tooth loss is about 7% in people aged 20 and over, and it reaches an even higher 23% in those aged 60 and over. One cohort suggested that tooth loss remarkably increased the risk of overall mortality and death from stroke, heart disease, and upper gastrointestinal cancer [3]. Another systematic review concluded that tooth loss is not only associated with increased incidence and prevalence of diabetes mellitus (DM) but also involved in DM-related diseases including heart disease, metabolic syndrome, and diabetic retinopathy [4]. However, whether tooth defects are related to the progression of IR, and if so, the underlying mechanism of this association remain elusive.

A growing body of research shows that diet quality has some correlation with tooth loss and IR. On the one hand, tooth loss, with its consequent reduction in masticatory function, inevitably affects eating habits and the intake of various nutrients, especially those that require tearing and grinding for chewing function [5,6]. On the other hand, dietary patterns play a crucial role in postprandial glycemic and insulin sensitivity [7]. Furthermore, a balanced diet would be an appropriate approach to prevent and treat IR and metabolic syndrome [8]. Given that diet quality is related to tooth loss and IR, we may hypothesize that tooth loss would induce IR through negatively affecting dietary intake.

Systemic immune-inflammation index (SII) is a good and stable indicator that could reflect the body’s local immune response and systemic inflammatory response [9,10]. It was proposed by Hu et al. in 2014 and has been extensively studied, which integrated platelets, neutrophils, and lymphocytes, and is calculated using platelet count× neutrophil count/lymphocyte count [11]. Chronic inflammation is one of the main causes of tooth loss, such as periodontal disease and caries, and the inflammatory state is not limited to the mouth but can also have an impact on the health of the whole body. A large number of researchers have worked to uncover the etiology and relationship between this chronic inflammatory dental disease and other diseases and have obtained important discoveries supporting an association between periodontal disease and systemic conditions such as T2DM, cardiovascular disease, adverse pregnancy outcomes, and osteoporosis [12]. Long-term infection during periodontitis can lead to exacerbated and dysregulated inflammatory responses, which in turn may contribute to poor control of blood glucose metabolism and increased insulin requirements [13]. Based on the above research findings, we hypothesize that tooth loss may induce IR by positively affecting SII.

Therefore, this study aims to explore the relationship between IR and tooth loss by analyzing cross-sectional study data from the National Health and Nutrition Examination Survey (NHANES) and investigate the mediating role of SII and diet quality level in it. The findings will provide a new theoretical and practical basis for the prevention and treatment of related diseases.

## 2. Method

### 2.1. Study Population

**Figure 1:**
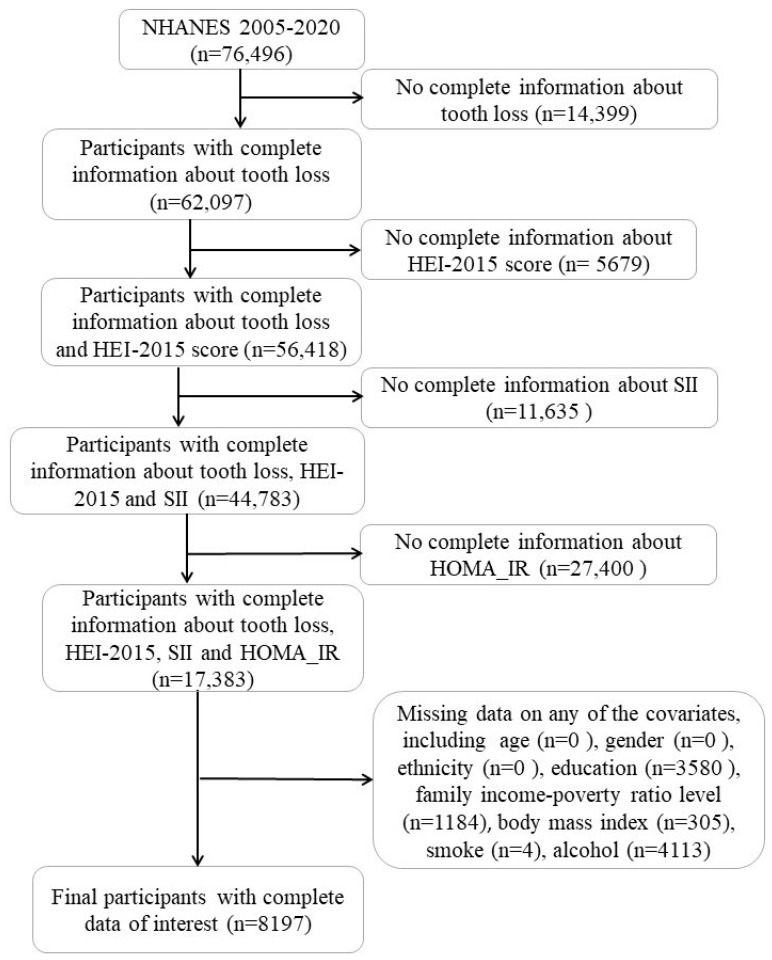
Participant Inclusion Flowchart.

This study used data from a publicly available database, NHANES, which is an ongoing cross-sectional survey that has collected data through interviews, examinations, and laboratory tests since 1999, with a sample size of approximately 5000 individuals each year. The study protocol was approved by the National Center for Health Statistics (NCHS) Research Ethics Review Board. All participants signed an informed consent at the time of recruitment. For the analysis in this report, we selected the 2005–2016 NHAHES data, which include a total of 76,496 individuals. Participants with incomplete information on tooth loss (14,399 participants), a HEI-2015 score (5679 participants), SII (11,635 participants), HOMA-IR (27,400 participants), and covariates (9186 participants) were excluded, and the final number of participants included in the study was 8197 (Figure 1).

### 2.2. Measures

For baseline data, means (±standard deviations, SDs) are used to describe continuous variables and counts (percentages) are used to describe categorical variables. A comparison of age continuous variables between the two groups was carried out by a *t*-test, and the chi-squared test was used to compare the demographic characteristics of subjects in the two IR groups. Subsequently, to assess the relationship between tooth loss and IR, diet quality and tooth loss and IR, and SII and tooth loss and IR, regression models were used. Finally, bootstrapping was applied to test the mediation effect of SII and diet quality between tooth loss and IR. The HEI-2015 score for assessing diet quality was calculated on the SAS 9.4, and other data were collated and analyzed in SPSS 18.0. A two-sided *p* < 0.05 was considered statistically significant.

Diet quality: The Healthy Eating Index (HEI) was used to assess diet quality. The HEI-2015 score, the government’s measure of adherence to dietary guidelines for Americans, includes 13 components (9 adequate food components and 4 moderate food components) [14]. The HEI-2015 score ranges from 0–100, with a higher HEI-2015 score indicating better diet quality. We used Day 1 Total Nutrient Intake (DR1TOT) to calculate the 13 components of the HEI-2015 score. A HEI-2015 score below 50, between 50 and 70, and above 70 are classified as inadequate, average, and best, respectively [15]. All participants were eligible for a diet quality assessment.

Tooth loss counts: In NHANES, complete oral health examinations are performed at a mobile examination center by trained and calibrated examiners. Tooth loss counts are recorded as the number of toothless parts. The complete dentition should contain 28 teeth, with the exception of the third molar, and the number of teeth lost in the complete dentition is recorded as zero. The number of teeth lost in patients with dentition defects is counted cumulatively in the edentulous part (meaning that there is no natural tooth or any form of restoration in that part) according to the situation of different dental positions.

SII: This combines three inflammatory cell types, namely, platelets, neutrophils, and lymphocytes, and is determined by multiplying the platelet counts by the neutrophil counts and dividing it by the lymphocyte counts.

The Homeostatic Model Assessment of IR (HOMA-IR): The HOMA-IR, proposed in 1985 by Matthews et al., is a method for quantifying IR by basal (fasting) glucose and insulin concentrations. The HOMA model has proven to be a powerful epidemiological and clinical tool for assessing IR and is widely used due to its simplicity and non-invasive nature. The formula involves multiplying the fasting insulin (μU/mL) by the fasting blood glucose (mmol/L) and dividing the result by 22.5. Under normal conditions, the HOMA-IR is less than 2.50, otherwise it can be judged as IR [16].

Covariates: All covariates include sex, age, ethnicity, education level, ratio of family income to poverty, body mass index (BMI), smoking status, and alcohol drinking. Sex was categorized as male and female. Race was classified into non-Hispanic white, non-Hispanic black, Mexican American, and other races [17]. Education level was classified as less than a high school diploma, high school graduate/GED, some college/AA degree, and college graduate or higher [18]. The ratio of family income to poverty was categorized as ≤100%, 100% to 300%, and >300%. BMI was calculated using measured height and weight as weight/height^2^ (kg/m^2^) and then categorized as ≤25, >25 to 30, and >30. Smoking behavior was assessed using a questionnaire on cigarette use. In the “smoking: cigarette use” questionnaire, respondents were asked if they had ever smoked cigarettes. Those who had smoked fewer than 100 cigarettes in their lifetime were classified as never smokers. Current smokers were defined as those who had smoked at least 100 cigarettes in their lifetime and were still smoking at the time of the questionnaire. Former smokers were those who had smoked at least 100 cigarettes in their lifetime but had quit by the time of the questionnaire. Smoking status was categorized into non-smoker, former smoker, and current smoker [19]. Alcohol drinking was categorized as ≥5 drinks/day and <5 drinks/day. A “drink” was defined as one-and-half ounces of liquor, a 5-ounce glass of wine, or a 12-ounce beer.

## 3. Result

### 3.1. Baseline Characteristics of the Studied Population

Table 1 summarizes the general characteristics of the population studied. The total number of subjects included was 8197, with 3861 individuals belonging to the IR group (HOMA-IR ≥ 2.5) and 4336 in the non-IR group (HOMA-IR < 2.5). In both analysis groups, there were significant differences between IR and non-IR individuals in age, sex, ethnicity, education level, household income/poverty ratio, BMI, and levels of smoking and alcohol consumption.

### 3.2. Association between Tooth Loss and HOMA-IR

In the linear regression models of HOMA-IR, the number of tooth loss was positively correlated with HOMA-IR (Table 2). Furthermore, HOMA-IR was divided into the non-IR group (HOMA-IR < 2.5) and the IR group (HOMA-IR ≥ 2.5), and a logistic regression model of HOMA-IR was established according to the number of tooth loss. Compared to the IR group without covariate adjustment, the risk of IR increased gradually with the number of teeth loss. After all covariates were adjusted, the fourth quartile of tooth loss was associated with an increase in HOMA-IR (Table 2, Model 2, OR = 1.301; 95% confidence interval (CI) = [1.102, 1.537]; *p* < 0.001) compared to the first quartile.

Compared with the first quartiles of tooth loss, the incidence of IR in the second, third, and fourth quartiles gradually increased. Unadjusted age, sex, ethnicity, education, family income/poverty ratio, BMI, smoking status and alcohol drinking level in the second, third, and fourth quartiles of tooth loss were 1.308 (95%CI = [1.127, 1.519]; *p* < 0.001), 1.366 (95%CI = [1.225, 1.524]; *p* < 0.001), and 1.646 (95%CI = [1.469, 1.845]; *p* < 0.001) times more likely to have HOMA_IR as compared to Q1, respectively. After adjusting all of the covariables, we observed that being in the third and fourth quartiles of tooth loss relative to the lowest were associated with 1.199 (95%CI = [1.048, 1.371]; *p* < 0.01) and 1.301 (95%CI = [1.102, 1.537]; *p* < 0.01) times higher odds of the incidence of insulin resistance.

### 3.3. Involvement of Diet Quality in the Association between Tooth Loss and HOMA-IR

The HEI-2015 score was inversely correlated with the number of tooth loss, and the highest quartile of the number of tooth loss was significantly correlated with the decrease in HEI-2015 score (β = −0.027, 95%CI = [−1.642, −0.092], *p* < 0.05, Table 3, Model 1). After adjusting for all covariates, the third and fourth quantiles of the number of tooth loss were correlated with the HEI-2015 score compared with the first quantile (Table 3, Model 2, Q3: β = −0.047, 95%CI = [−2.203, −0.699], *p* < 0.001; Q4: β = −0.121, 95%CI = [−4.839, −2.974], *p* < 0.001).

Diet quality has a significant influence on the IR group (HOMA-IR ≥ 2.5) based on the regression analysis result. In the linear regression model without covariate correction, compared to average diet quality, best diet quality has a positive effect on HOMA-IR (β = −0.025; 95%CI = [−1.182, 0.062]; *p* < 0.05, Table 4, Model 1); inadequate diet quality has a negative effect (β = 0.052; 95%CI = [0.408, 1.039]; *p* < 0.001, Table 4, Model 1) on the contrary. Furthermore, with the adjustment of all covariates in the logistic regression model, compared to average diet quality, best diet quality is a protective factor for HOMA-IR (Table 4, Model 2, OR = 0.776; 95%CI = [0.641, 0.939]; *p* < 0.01); inadequate diet quality is a risk factor (Table 4, Model 2, OR = 1.267; 95%CI = [1.138, 1.411]; *p* < 0.001) conversely.

### 3.4. Involvement of SII in the Association between Tooth Loss and HOMA-IR

The amount of tooth loss had a positive effect on SII. According to the linear regression model, the highest number of tooth loss has a significant effect on SII (Table 5, Model 1, β = 0.072; 95%CI = [36.135, 72.336]; *p* < 0.001) compared to the case without tooth loss. After the adjustments for covariates, the highest number of tooth loss was found to have a significant effect on SII (Table 5, Model 2, β = 0.032; 95%CI = [1.777, 47.448]; *p* < 0.05).

SII has a significant positive effect on HOMA-IR. In the logistic regression model with adjusted covariates, it can be seen that compared with the first quantile of SII, the highest score is significantly correlated with the higher incidence of IR (Table 6, Model 2, OR = 1.363; 95%CI = [1.179, 1.575]; *p* < 0.001).

### 3.5. Analysis of Mediating Effects of Diet Quality and SII in the Association between Tooth Loss and HOMA-IR

**Figure 2:**
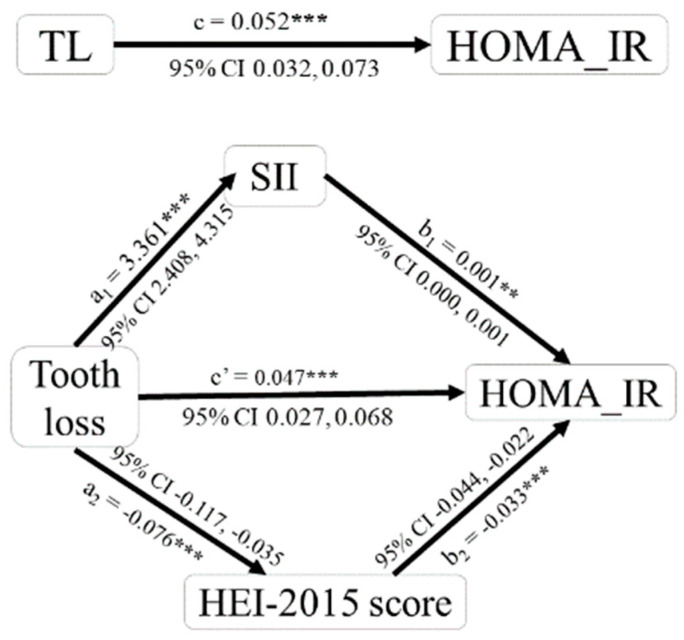
Partial mediation of HEI-2015 score and SII in the association between tooth loss and HOMA-IR. **: *p* < 0.01, ***: *p* < 0.001.

Through the mediation effect analysis, this study further validates the influence of both diet quality and SII in the association between HOMA-IR and tooth loss, so the model was refined (Figure 2). Based on the result (Table 7), we can see that diet quality and SII play a partial mediating role in the relationship between HOMA-IR and tooth loss, and the mediating effect ratio for the total effect value is 4.731% and 4.576%, respectively. The mediating effect of diet quality and SII in the relationship between HOMA-IR and tooth loss both is 0.003 (95%CI = [0.001, 0.004]).

## 4. Discussion

In this study, we found that tooth loss was correlated with IR, and SII and diet quality were examined as mediators of this association. These findings shed light on the link between tooth loss and IR and the pathways it affects, namely, that severe tooth loss can lead to a decrease in the quality of the diet and an increase in SII, and highlight the importance of a timely diagnosis and repair for patients with dentition defects or loss. Meanwhile, patients with long-term severe tooth loss are advised to be aware of the occurrence of IR and related diseases. This study provides an important reference for further exploration of the relationship between dental health and general health.

As is well known, the main causes of tooth loss are severe dental caries and periodontitis. Meanwhile, the relationship between diabetes and periodontitis is two-sided [20]. On the one hand, individuals with progressive periodontal disease may have difficulty regulating their blood sugar. On the other hand, diabetes is a leading cause of periodontitis and subsequent tooth loss [21,22], and the presence of diabetes may result in more than nine additional missing teeth [23]. However, it is unclear whether tooth loss has an effect on the development of diabetes. In this study, the relationship between IR and tooth loss, one of the core pathological mechanisms of T2DM, was confirmed using linear and logistic regression. It has previously been shown that tooth loss can induce IR in a dose-dependent manner, that is, the risk of IR increases with the number of teeth lost and thus with the risk of metabolic diseases such as T2DM. Based on this, effective measures such as promoting oral health, preventing tooth loss, and repairing lost teeth should be taken to prevent adverse effects on overall health. But it is not enough, and it is necessary to find the mechanism of the relationship between IR and tooth loss to deepen our understanding of the influence of tooth loss on overall health and guide us to develop more targeted strategies to compensate for the negative effects of tooth loss.

To further explore the possible mechanisms of the relationship between IR and tooth loss, the mediating effect of diet quality was firstly demonstrated in this study. The mediating effect ratio for the HEI-2015 score was 4.731%. Recent findings strongly suggest that oral health may be indicative of general health [12]. Tooth loss causes a decline in oral chewing function, which changes the patient’s original eating pattern, reducing the quality of the diet and thus inducing IR. Previous studies have established that people with severe tooth loss may have reduced their intake of beans, vegetables, and fruits, indicating a lack of fiber, micro-elements, and vitamins due to insufficient chewing function [24,25,26]. In addition, poor dietary quality, an imbalanced diet including the insufficient intake of fruits and vegetables, and inadequate dietary variety were significantly associated with an increased risk of pre-diabetes [27]. An observational study conducted on a cohort of individuals over a period of 4 years found that an enhancement in overall diet quality was linked to a decreased risk of developing type 2 diabetes mellitus (T2DM) during the subsequent 4 years. In turn, decreased diet quality was associated with an increased risk of T2DM [28]. Thus, our hypothesis can be confirmed that tooth loss induces IR by reducing the quality of a patient’s diet. And the greater the number of missing teeth, the more marked the decline in the quality of the diet, and consequently, the greater the negative effect.

At the same time, SII as another mediating factor is validated by the mediating effect. The mediating effect ratio for SII was 4.576%. Tooth loss is the result of periodontitis, which is the main cause of tooth loss in adults. Periodontitis is characterized by inflammation, and evidence suggests that it is a contributing factor to systemic inflammation, which may be reduced following periodontal treatment [29,30]. Chronic periodontitis is believed to elevate the risk of diabetes by promoting infection and inflammation [13,31]. Periodontal therapy, on the other hand, contributes to improved insulin sensitivity and glycemic control by reducing periodontal inflammation and blood levels of cytokines and other inflammatory mediators [32]. Furthermore, Bui et al. confirmed that, secondary to oral infection, *T. denticola* and *P. gingivalis* are capable of triggering a systemic immune response [33]. SII has been acknowledged as a novel, reliable, and consistent marker that captures both localized immune responses and systemic inflammation across the entire human body [34]. SII has been found to be linked to an increased risk of periodontitis [35,36]. A study proves that elevated levels of inflammation markers and mediators, such as C-reactive protein, fibrinogen, plasminogen activator inhibitor-1, IL-6, sialic acid, and white cell count, correlate with the incidence of T2DM. Furthermore, it has been shown that TNF-α, a pro-inflammatory cytokine, plays a crucial role in inducing IR, thereby connecting inflammation to the pathogenesis of IR [37]. In the context of diabetes, inflammation is one of the key underlying factors, with low-grade systemic inflammation being an early and primary pathological event that contributes to the development of IR in various insulin-sensitive tissues [38,39,40]. Another important mechanism by which inflammation leads to IR is the activation of Toll-like receptors (TLRs), which are part of the innate immune response and responsible for pattern recognition. Interestingly, different rodent models with TLR4 ablation, either via genetic knockout or pharmacological inhibition, have been shown to prevent IR [41,42,43]. In summary, our alternative hypothesis has been confirmed that SII is an important mediator of IR induced by missing teeth.

As a part of the human body, the role that teeth may play in overall health cannot be ignored. In the face of tooth loss, on the one hand, we should actively control the etiology and timely repair the missing teeth to maintain the integrity and stability of the function of the stomatognathic system. On the other hand, for patients with persistent or severe tooth loss, we should consider whether poor oral health has affected general health. We should actively prevent and screen for the occurrence of related diseases, such as those associated with IR as confirmed by this study. Once identified, we should intervene as soon as possible to avoid the serious consequences of its continued malignant development.

This study is the first to confirm the relationship between tooth loss and IR in a large, nationally representative sample and to propose a mediating role for diet quality and SII. However, there are some limitations to this study. Firstly, the main limitation of this study is the data collection method and cross-sectional design, which hinder the exploration of causality. Future research will use the longitudinal study design to further validate the results. Secondly, this study only considered the number of tooth loss and did not consider the location of the tooth loss, which may affect the dietary status. In addition, it is unknown whether timely restoration after tooth loss and different denture restoration methods will offset to some extent the negative effects of tooth loss on IR. Thirdly, the low mediating effect of diet quality and SII on the relationship between tooth loss and IR suggests that other possible pathways still exist. In the future, we will design prospective cohort studies to remedy the aforementioned shortcomings, conduct more in-depth and detailed validation of the results of this study, and explore more possible mediating mechanisms.

## 5. Conclusions

Our study revealed the relationship between IR and tooth loss and further explored the mediating role of diet quality and SII in the number of missing teeth and IR, emphasizing that improving diet quality and reducing SII can effectively prevent and treat IR and related diseases. It provides new theoretical support for the study of IR mechanisms and new ideas and approaches for the prevention and treatment of related disorders.

## Figures and Tables

**Table 1:** Characteristics of study population, NHANES, USA, 2005–2020.

Characteristics	Total (*n* = 8197)	HOMA-IR < 2.5 (*n* = 4336)	HOMA-IR ≥ 2.5 (*n* = 3861)	*p*-Value
Age	47.33 ± 0.18	45.96 ± 0.26	48.87 ± 0.26	<0.001
Sex				<0.001
Female	3761 (45.9)	2106 (48.6)	1655 (42.9)	
Male	4436 (54.1)	2230 (51.4)	2206 (57.1)	
Race and ethnicity				<0.001
Non-Hispanic White	3981 (48.6)	2282 (52.6)	1699 (44.0)	
Non-Hispanic Black	1659 (20.2)	833 (19.2)	826 (21.4)	
Mexican American	1097 (13.4)	434 (10.0)	663 (17.2)	
Other	1460 (17.8)	787 (18.2)	673 (17.4)	
Education level				<0.001
Less than high school	1440 (17.6)	697 (16.1)	743 (19.2)	
High school or equivalent	1847 (22.5)	908 (20.9)	939 (24.3)	
College or above	4910 (59.9)	2731 (63.0)	2179 (56.5)	
Ratio of family income to poverty				<0.001
0–1.0 (including 1.0)	1380 (16.8)	703 (16.2)	677 (17.5)	
1.0–3.0(including 3.0)	3172 (38.7)	1578 (36.4)	1594 (41.3)	
>3.0	3645 (44.5)	2055 (47.4)	1590 (41.2)	
Body mass index				<0.001
Normal (<25.0)	2626 (32.0)	2145 (49.5)	481 (12,5)	
Overweight (25.0–30.0)	2922 (35.6)	1576 (36.3)	1346 (34.8)	
Obese (>30.0)	2649 (32.3)	615 (14.2)	2034 (52.7)	
Smoking status				<0.001
Non-smoker	4148 (50.6)	2204 (50.8)	1944 (50.3)	
Former smoker	2159 (26.3)	1048 (24.2)	1111 (28.8)	
Current smoker	1890 (23.1)	1084 (25.0)	806 (20.9)	
Alcohol drinking				<0.05
≥5 drinks/day	1159 (14.1)	574 (13.2)	585 (15.2)	
<5 drinks/day	7038 (85.9)	3762 (86.8)	3276 (84.8)	

*t*-test was used to compare continuous variables; chi-squared test was used to compare the demographic characteristics.

**Table 2:** Regression analysis of the association between tooth loss and HOMA-IR.

Number of Tooth Loss	Linear Regression Analysis		Logistic Regression Analysis			
			Model 1		Model 2	
	β (95%CI)	*p*-Value	OR (95%CI)	*p*-Value	OR (95%CI)	*p*-Value
Continuous	0.052 (0.031, 0.073)	<0.001	1.018 (1.012, 1.025)	<0.001	1.013 (1.005, 1.022)	<0.01
Q1 (0)	Reference	-	Reference	-	Reference	-
Q2 (1)	0.023 (0.008, 1.035)	<0.05	1.308 (1.127, 1.519)	<0.001	1.078 (0.906, 1.282)	0.396
Q3 (2–5)	0.031 (0.107, 0.859)	<0.05	1.366 (1.225, 1.524)	<0.001	1.199 (1.048, 1.371)	<0.01
Q4 (6–28)	0.073 (0.802, 1.584)	<0.001	1.646 (1.469, 1.845)	<0.001	1.301 (1.102, 1.537)	<0.01

Model 1: Logistic regression model of IR group (HOMA-IR ≥ 2.5) according to the number of tooth loss without covariate correction. Model 2: Logistic regression model of IR group (HOMA-IR ≥ 2.5) according to the number of tooth loss after being adjusted for age, sex, ethnicity, education, family income/poverty ratio, BMI, smoking status, and alcohol drinking level. Q1: 1st quartile; Q2: 2nd quartile; Q3: 3rd quartile; Q4: 4th quartile.

**Table 3:** Linear regression models of HEI-2015 score established based on the quartiles of the number of tooth loss.

Number of Tooth Loss	β (95%CI)			
	Model 1	*p*-Value	Model 2	*p*-Value
Continuous	−0.040 (−0.116, −0.035)	<0.001	−0.112 (−0.258, −0.164)	<0.001
Q1 (0)	Reference	-	Reference	-
Q2 (1)	0.017 (−0.249, 1.786)	0.139	−0.005 (−1.179, 0.780)	0.689
Q3 (2–5)	0.014 (−0.299, 1.191)	0.240	−0.047 (−2.203, −0.699)	<0.001
Q4 (6–28)	−0.027 (−1.642, −0.092)	<0.05	−0.121 (−4.839, −2.974)	<0.001

Model 1: No covariate correction. Model 2: Adjusted for age, sex, ethnicity, education, family income/poverty ratio, BMI, smoking status, and alcohol drinking level. Q1: 1st quartile; Q2: 2nd quartile; Q3: 3rd quartile; Q4: 4th quartile.

**Table 4:** Regression analysis of the association between HEI-2015 score and HOMA-IR.

HEI-2015 Score	Model 1		Model 2	
	β (95%CI)	*p*-Value	OR (95%CI)	*p*-Value
Continuous	0.040 (0.000, 0.001)	<0.001	0.986 (0.982, 0.990)	<0.001
A1 (average)	Reference	-	Reference	-
A2 (best)	−0.025 (−1.182, 0.062)	<0.05	0.776 (0.641, 0.939)	<0.01
A3 (inadequate)	0.052 (0.408, 1.039)	<0.001	1.267 (1.138, 1.411)	<0.001

Model 1: linear regression model of HOMA-IR according to three categories of HEI-2015 score without covariate correction. Model 2: logistic regression model of IR group (HOMA-IR ≥ 2.5) according to three categories of HEI-2015 score after being adjusted for age, sex, ethnicity, education, family income/poverty ratio, BMI, smoking status, and alcohol drinking level. A1: average diet quality (50 ≤ HEI-2015 score ≤ 70); A2: best diet quality (70 < HEI-2015 score); A3: inadequate diet quality (HEI-2015 score < 50).

**Table 5:** Linear regression models of SII established based on the quartiles of the number of tooth loss.

Number of Tooth Loss	β (95%CI)			
	Model 1	*p*-Value	Model 2	*p*-Value
Continuous	0.076 (2.408, 4.315)	<0.001	0.051 (1.121, 3.406)	<0.001
Q1 (0)	Reference	-	Reference	-
Q2 (1)	0.006 (−17.623, 29.890)	0.613	−0.006 (−30.274, 17.710)	0.608
Q3 (2–5)	0.032 (5.485, 40.268)	<0.05	0.010 (−11.046, 25.804)	0.432
Q4 (6–28)	0.072 (36.135, 72.336)	<0.001	0.032 (1.777, 47.448)	<0.05

Model 1: No covariate correction. Model 2: Adjusted for age, sex, ethnicity, education, family income/poverty ratio, BMI, smoking status, and alcohol drinking level. Q1: 1st quartile; Q2: 2nd quartile; Q3: 3rd quartile; Q4: 4th quartile.

**Table 6:** Regression analysis of the association between SII and HOMA-IR.

SII	Model 1		Model 2	
	β (95%CI)	*p*-Value	OR (95%CI)	*p*-Value
Continuous	0.040 (0.000, 0.001)	<0.001	1.000 (1.000, 1.001)	<0.001
Q1 (≤325.18)	Reference	-	Reference	-
Q2 (325.19–452.57)	0.027 (0.003, 0.851)	<0.05	1.130 (0.980, 1.303)	0.092
Q3 (452.58–640.40)	0.037 (0.168, 1.015)	<0.01	1.234 (1.070, 1.423)	<0.01
Q4 (≥640.40)	0.060 (0.542, 1.389)	<0.001	1.363 (1.179, 1.575)	<0.001

Model 1: linear regression model of HOMA-IR according to SII without covariate correction. Model 2: logistic regression model of IR group (HOMA-IR ≥ 2.5) according to SII after being adjusted for age, sex, ethnicity, education, family income/poverty ratio, BMI, smoking status, and alcohol drinking level. Q1: 1st quartile; Q2: 2nd quartile; Q3: 3rd quartile; Q4: 4th quartile.

**Table 7:** Mediation analyses of dietary quality and SII in the association between tooth loss and HOMA-IR.

	Tooth Loss →HEI-2015 Score → HOMA-IR	Tooth Loss → SII → HOMA-IR
C (total effect)	0.052 ***	0.052 ***
a	−0.076 ***	3.361 ***
b	−0.033 ***	0.001 **
a × b (mediating effect)	0.002	0.002
a × b (BOOT SE)	0.001	0.001
a × b (z-value)	3.089	2.846
a × b (*p*-value)	0.002	0.004
a × b (95% Boot CI)	0.001, 0.004	0.001, 0.004
c’ (direct effect)	0.047 ***	0.047 ***
Effect ratio	4.731%	4.576%
Test conclusion	Partial mediation	Partial mediation

Note: **: *p* < 0.01; ***: *p* < 0.001.

## Data Availability

Data are contained within the article.
